# Comprehensive Review of Antiphospholipid Syndrome: Over Four Decades of Advances and Challenges

**DOI:** 10.3390/cells15040356

**Published:** 2026-02-17

**Authors:** Takao Koike

**Affiliations:** 1 Graduate School of Medicine, Hokkaido University, N-15 W-7, Kita-ku, Sapporo 060-8638, Japan; tkoike@med.hokudai.ac.jp or takaosapporo@gmail.com; Tel.:+81-9088991729; 2 Hokkaido Medical Center for Rheumatic Diseases, Kotoni 1-13, Nishi-ku, Sapporo 063-0811, Japan

**Keywords:** antiphospholipid syndrome (APS), β2-glycoprotein I (β2GPI), anticardiolipin antibody (aCL), antiprothrombin antibody (aPS/PT), anti-β2GPI domain I antibody, antiphospholipid score, classification criteria, two-hit hypothesis, neutrophil extracellular traps (NETs), interferon signature

## Abstract

Antiphospholipid syndrome (APS), first described in 1983, is a systemic autoimmune disorder characterized by recurrent arterial and venous thrombosis, pregnancy complications, and persistent antiphospholipid antibodies (aPL). Over four decades, significant advancements have been made in understanding APS pathogenesis, diagnostics, and treatment. Key discoveries include the development of standardized anticardiolipin antibody (aCL) assays, the identification of β2-glycoprotein I (β2GPI) as a critical cofactor, and the elucidation of the “two-hit” hypothesis, which explains thrombotic events through a combination of aPL-induced prothrombotic priming and secondary external triggers. Recent research has highlighted the roles of complement activation, neutrophil extracellular traps (NETs), and genetic predispositions shared with systemic lupus erythematosus (SLE). Innovations like the antiphospholipid score (aPL-S) and updated classification criteria, including the 2023 ACR/EULAR guidelines, have improved diagnostic precision and risk stratification. Despite these advances, challenges remain in assay standardization and addressing seronegative APS. Future directions emphasize the integration of multimodal biomarkers, precision diagnostics, and targeted therapies aimed at complement and NET pathways. These efforts aim to achieve individualized care, improving outcomes for APS patients through harmonized diagnostics, mechanistic therapeutics, and data-driven approaches. This review underscores the evolving understanding of APS and its potential for personalized management strategies.

## 1. Introduction

Antiphospholipid syndrome (APS), first described by Graham Hughes in 1983, represents a prototypical autoimmune thromboinflammatory disorder characterized by recurrent arterial and venous thrombosis, pregnancy morbidity, and persistent antiphospholipid antibodies (aPL) [1].

This comprehensive review synthesizes four decades of APS research, with particular emphasis on our laboratory’s foundational and ongoing contributions to the field. Our work has spanned the development of standardized anticardiolipin enzyme-linked immunosorbent assay (ELISA) methodology [2], discovery of β2-glycoprotein I (β2GPI) as a critical cofactor [3,4,5,6,7], discovery of animal model of APS [8], characterization of antiprothrombin antibodies and lupus anticoagulant (LA) mechanisms [9,10,11,12,13,14], elucidation of cellular signaling pathways [15,16,17], investigation of complement activation [18,19,20,21], and translational advances toward precision diagnostics and targeted therapeutics [22,23,24]. These contributions are presented within the context of the broader international APS research community to provide a comprehensive understanding of disease pathogenesis and clinical management.

Since our department has conducted minimal research on pregnancy-related complications of APS, this review does not extensively address this topic. I would also like to note in advance that the latest therapeutic developments in APS management are not covered in the present review.

## 2. Interpreting APS Through Its History

### 2.1. From Biological False Positives to Standardized Detection

The origins of APS investigation trace to observations in systemic lupus erythematosus (SLE) patients who exhibited biological false-positive serologic tests for syphilis, leading to the recognition that these patients harbored antibodies reactive in phospholipid-dependent assays [25,26]. These early findings established aPL as a clinically meaningful biomarker associated with thrombotic complications in autoimmune disease.

A critical milestone came in 1984 when the ELISA for anticardiolipin antibodies (aCL) was developed, providing the first standardized method for aPL detection [2]. This technical advance enabled systematic characterization of APS as a distinct clinical entity and facilitated international collaborative research. Subsequent studies revealed that aCL displays broad cross-reactivity with negatively charged phospholipids, including phosphatidylserine and phosphatidylglycerol, and double-stranded DNA expanding the antigenic landscape beyond cardiolipin and prompting investigation into the true molecular targets underlying pathogenic antibody binding [27,28,29,30].

### 2.2. Discovery of β2GPI as Essential Cofactor

The field was transformed in 1990 with the identification of β2-glycoprotein I (β2GPI) as a necessary cofactor for aCL binding, fundamentally reframing APS pathogenesis around this phospholipid-binding protein [3,31,32]. β2GPI is a 50 kDa plasma glycoprotein composed of five short consensus repeat (SCR; also termed “Sushi”) domains, with domain V conferring specific affinity for anionic phospholipid membranes. This discovery shifted the focus from phospholipids themselves to protein-phospholipid complexes as the primary antigenic targets in APS [33,34].

Subsequent structural studies supported a conformational switch model wherein β2GPI exists in a compact, closed conformation in solution but adopts an extended, open configuration upon binding to negatively charged membrane surfaces [4,5,35].

**Figure 1:**
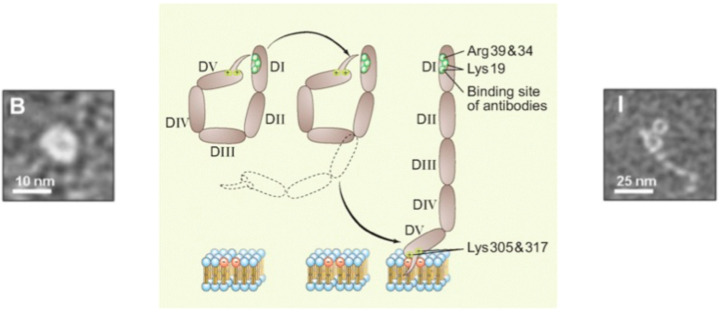
Structural basis for antibody recognition: the conformational plasticity of β2GPI [Adapted from reference [37]]. B: Circular form and I: Enlarged form.

Crystallographic and solution studies have mapped specific epitope regions within β2GPI that are critical for pathogenic antibody recognition [36,37] (Figure 1).

### 2.3. β2GPI: Functional Diversity Beyond Antigenicity

Domain 5 of β2GPI mediates diverse protective functions, including anticoagulation, complement regulation, apoptotic cell clearance, and interactions with anionic phospholipids and multiple receptors (e.g., ApoER2/LRP8, annexin A2, TLR2/4). Redox-sensitive allosteric disulfide bonds in D5 (Cys288–Cys326) modulate membrane binding and autoantibody recognition; the reduced (free thiol) form appears cytoprotective, whereas nitration correlates with thrombosis risk in APS [34].

Post-translational modifications of β2GPI significantly contribute to antiphospholipid syndrome. Phosphorylation, oxidation, and proteolytic cleavage enhance β2GPI immunogenicity, promoting autoantibody formation. N-glycosylation alterations and thiol-disulfide exchange modify antibody recognition and expose cryptic epitopes. AGE modification increases thrombotic risk via inflammation. These modifications drive autoantibody production and strengthen β2GPI interactions with endothelial cells and platelets, representing potential therapeutic targets [38,39,40].

The pathophysiology of APS can also be explained by the interplay between impaired fibrinolysis and β2GPI [17].

As mentioned, β2GPI exhibits remarkable functional diversity beyond serving as an autoantigen. Plasmin-mediated cleavage between Lys-317 and Thr-318 generates “nicked” β2GPI, which loses phospholipid-binding capacity but gains the ability to bind plasminogen and suppress plasmin generation, thereby modulating fibrinolytic activity [41]. Furthermore, nicked β2GPI interacts with angiostatin4.5, suggesting roles in angiogenesis regulation and vascular remodeling in the context of thrombotic disease [42]. These findings illustrate the complex, context-dependent functions of β2GPI in hemostasis and vascular biology.

Genetic studies have identified β2GPI polymorphisms as important contributors to APS susceptibility. The Val/Leu247 variant in domain V alters the protein’s tertiary structure, increasing its antigenicity and favoring the development of anti-β2GPI antibodies, particularly in patients with primary APS [43]. These findings demonstrate how inherited structural variations can influence autoantigen presentation and subsequent immune recognition.

### 2.4. Shared Genetic Architecture with SLE

Population genetic studies have revealed significant overlap between APS and SLE genetic risk factors. Increased frequencies of STAT4 single-nucleotide polymorphisms have been identified in Japanese cohorts with both SLE and APS, along with associations involving BANK1, BLK, and the 1q25.1 chromosomal region. These findings underscore shared immunoregulatory pathways that influence autoantibody generation, immune activation, and thrombotic predisposition across the lupus disease spectrum. As discussed later, the relationship between the interferon signature and APS closely mirrors that observed in SLE, and the resulting dynamics of adaptive immunity are likewise highly similar; thus, APS may represent an SLE-spectrum phenotype rather than a separate entity [23,44]. Taiwanese nationwide data strongly bolster our view, showing that patients with APS have nearly an 80-fold increased risk of developing SLE, with most SLE diagnoses occurring within five years [45].

### 2.5. APS/Lupus-Prone Mouse Model

NZW × BXSB F1 mice, which spontaneously develop lupus-like disease with myocardial infarction and produce both aCL and anti-β2GPI antibodies, have been instrumental in elucidating APS pathophysiology [8]. Monoclonal antibodies derived from these animals, WBCAL1, exhibit specificity patterns remarkably similar to human APS antibodies.

### 2.6. Monoclonal Standards Advancing APS Serology

International standardization of aCL and anti-β2GPI assays has progressed through two monoclonal standards: HCAL and EY2C9 [46,47], which reduce inter-laboratory variability and improve APS serology reliability. HCAL provides the IgG standard. It is a human–mouse chimeric antibody engineered by grafting WBCAL1’s murine variable regions onto human constant regions; WBCAL1 originates from NZW × BXSB mice prone to APS-like autoimmunity, as described above. HCAL shows β2GPI-dependent binding and mimics key properties of patient autoantibodies, enabling robust calibration for IgG-class aCL/anti-β2GPI assays [46]. EY2C9 serves as the IgM standard. Derived from an APS patient’s peripheral blood B cells, it binds β2GPI with high affinity and mirrors IgM reactivity in APS sera, supporting accurate quantitation across platforms [47].

Monoclonal standards address lot instability and site-to-site variability seen with traditional calibration. Using HCAL and EY2C9 enables reproducible, scalable calibration curves with defined specificity and affinity, improving analytical precision, harmonization across methods and manufacturers, and diagnostic accuracy and risk stratification—especially when combined with β2GPI-dependent and domain-specific assays [48].

### 2.7. Antiprothrombin Antibodies and Coagulation Interference

Antiprothrombin antibodies, another important component of the aPL repertoire, bind to prothrombin when exposed on immobilized phosphatidylserine surfaces (phosphatidylserine-dependent antiprothrombin antibodies (aPS/PT)), recognizing neo-epitopes generated by conformational changes in the protein [9].

Monoclonal aPS/PT antibody 231D exhibits robust LA activity and potently inhibits thrombin generation under defined experimental conditions [13]. These studies emphasize the importance of assay context, antigen conformation, and phospholipid presentation in detecting and interpreting LA phenomena, providing mechanistic insight into how aPS/PT disrupts normal coagulation cascades [10,11,12,13,14].

aPS/PT correlate strongly with LA activity [9,10] and, when combined with anti-β2GPI domain I antibodies (as described later), can significantly improve diagnostic accuracy and risk stratification, particularly in patients with ambiguous serologic profiles or apparent seronegative disease. These developments support a multimarker diagnostic approach that reflects the antigenic heterogeneity of APS.

### 2.8. Anti-β2GPI Domain I Antibodies

Antibodies targeting Domain I (DI) of β2GPI represent a high-risk subset within aPL and are strongly linked to the clinical phenotype of APS [49,50,51]. β2GPI comprises five domains; DI harbors the principal pathogenic epitope, centered around the R39–R43 region, which becomes exposed upon binding to negatively charged phospholipids. DI-specific antibodies show the most robust association with venous and arterial thrombosis and with obstetric complications, including early miscarriage, placental dysfunction, fetal growth restriction, and preeclampsia. Their prevalence is higher in “triple-positive” patients (LA, aCL, anti-β2GPI), and DI positivity can refine risk stratification beyond conventional aPL assays [37,52].

Assay development has focused on ELISA platforms using immobilized DI, with peptide-based or competitive formats targeting the R39–R43 epitope. However, inter-assay variability, antigen conformation, isotype handling (IgG/IgM/IgA), and cutoff standardization remain challenges, limiting routine implementation. Currently, DI testing is best viewed as an adjunct for identifying high-risk APS patients, complementing the established triad of tests.

## 3. Enhanced Risk Stratification

### 3.1. The Influence of Gender

Male APS patients demonstrate significantly higher rates of arterial thrombotic complications, with myocardial infarction showing an odds ratio of 3.77 (95% CI 1.09–13.08) and limb ischemia occurring in 19% versus 1% in females. Male patients display elevated IgM anticardiolipin titers (26.5% vs. 9.2%, *p* = 0.004), reflecting more aggressive disease phenotypes. Male gender emerged as an independent risk factor for thromboembolic recurrence (hazard ratio 3.699, 95% CI 1.659–8.246). Gender disparities involve higher smoking (62% vs. 8%) and hyperhomocysteinemia (32% vs. 12%), alongside X-linked immune regulatory genes and gonadal steroid hormone effects. These findings support gender-stratified risk assessment and personalized management strategies [53].

### 3.2. The Antiphospholipid Score (aPL-S)

We introduced the antiphospholipid score (aPL-S) as a quantitative tool for assessing APS probability and predicting thrombotic risk [22]. By integrating multiple aPL tests (IgG/IgM aCL, IgG/IgM anti-β2GPI, LA (5 clotting assays) and IgG/IgM aPS/PT), we developed a comprehensive scoring system that reflects each patient’s aPL profile. In retrospective studies of 233 and 411 patients with autoimmune diseases, higher aPL-S scores strongly correlated with thrombosis and pregnancy morbidity, with scores exceeding 30 significantly increasing thrombotic risk.

The aPL-S represents a methodological advance over existing categorical diagnostic approaches. Unlike the revised Sapporo criteria [54], which employ binary classification, our quantitative scoring system better captures the spectrum of aPL positivity. We demonstrated superior diagnostic and predictive accuracy compared to categorical revised Sapporo classification using ROC curve analysis. Although the partial aPL-S has clinical utility, the complete aPL-S—which includes anti-PS/PT antibodies—showed significantly superior predictive value for thrombosis [22]. In this study, “partial aPL-S” refers to the reduced version of the antiphospholipid score that is calculated using only a subset of antiphospholipid antibodies (IgG/IgM aCL, IgG/IgM anti-β2GPI, and LA (2 clotting assays)), as defined and analyzed in Otomo et al. [22].

Our approach extends prior observations by Pengo and colleagues, who reported that triple positivity for aCL, LA, and anti-β2GPI conferred higher thrombotic risk [52]. However, Pengo’s analysis employed categorical triple positivity assessment, whereas our aPL-S introduces continuous quantification, enabling more refined risk stratification and individualized therapeutic decisions.

These findings suggest the aPL-S could guide personalized antithrombotic therapy based on quantitative risk profiles. While prospective validation studies remain necessary, this quantitative biomarker represents an important advancement toward precision medicine in APS management.

### 3.3. GAPSS

The Global Antiphospholipid Syndrome Score (GAPSS) is a weighted algorithm combining traditional cardiovascular risk factors (hypertension, hyperlipidemia) with aPL profile—LA, aCL, anti-β2GPI, and aPS/PT—to estimate the risk of thrombotic and obstetric events in APS, SLE, and aPL carriers [55,56].

To enhance feasibility and generalizability, an adjusted version (aGAPSS) was developed excluding aPS/PT [57]. The principal reasons were limited assay availability, lack of inter-laboratory standardization, and higher analytical variability of aPS/PT at the time of derivation/validation, which could compromise reproducibility and hinder adoption across centers. By retaining the best-standardized tests (LA, aCL, anti-β2GPI) plus traditional risk factors, aGAPSS preserved or improved predictive performance and calibration in external cohorts while enabling broader clinical use [57]. Subsequent studies confirmed that aGAPSS stratifies recurrence risk and supports individualized prophylaxis, though prospective, multi-ethnic validation and potential recalibration remain advisable as assay technologies evolve and as the clinical role of aPS/PT continues to be clarified.

### 3.4. Morbidity and Mortality in APS Based on Cluster Analysis

This study, conducted by our group at Hokkaido University Hospital, is the first to apply cluster analysis for risk stratification in Japanese patients with APS [24]. By analyzing clinical and serological data from 168 patients over a 10-year period, we identified three distinct subgroups: Cluster A (secondary APS, primarily associated with SLE), Cluster B (accumulation of cardiovascular risk factors and arterial thrombosis), and Cluster C (triple-positive aPL and venous thrombosis). Among these, Cluster B demonstrated significantly worse outcomes, with the highest event rate (5.56 per 100 patient-years) and mortality rate (1.59 per 100 patient-years) compared to the other clusters.

Our findings highlight the critical role of cardiovascular risk factors in determining the prognosis of APS patients, particularly in those with arterial thrombosis. The study also underscores the importance of managing vascular risk factors and developing tailored therapeutic strategies based on risk stratification. By integrating both clinical and serological data, our novel approach provides a more comprehensive understanding of APS heterogeneity and may serve as a foundation for future studies and personalized treatment strategies. This work emphasizes the potential of cluster analysis in improving the management of complex autoimmune diseases like APS.

### 3.5. Evolution of Classification Criteria

#### From Sapporo to Sydney

The 1998 Sapporo classification criteria for APS emerged from an international effort to standardize diagnosis in a heterogeneous condition linked to thrombosis and pregnancy morbidity [58]. Prior to Sapporo, diverse clinical presentations and variable laboratory assays—aCL, LA, and anti-β2GPI—led to inconsistent case definitions and research comparability problems. Convened at the VIII International Symposium on Antiphospholipid Antibodies in Sapporo, experts synthesized evidence to define combined clinical and laboratory requirements, including persistent antibody positivity on two occasions at least 6 weeks apart. The criteria aimed to harmonize patient selection for studies, improve prognostic stratification, and guide therapeutic research. They later informed the 2006 revised (Sydney) criteria.

The 2006 Sydney revision aimed to improve APS classification reliability and global consistency [54]. It incorporated accumulated evidence since the Sapporo criteria to refine which antibodies to test, when, and how. Key changes standardized laboratory methods (especially LA), required medium/high titers and persistent positivity confirmed at least 12 weeks apart to reduce transient or low-titer false positives, and emphasized anti-β2GPI alongside aCL. Clinical event definitions for thrombosis and pregnancy morbidity were clarified with imaging/pathology confirmation to enhance comparability across studies. Importantly, the revision reasserted that these are research “classification” criteria, not bedside “diagnostic” rules, guiding uniform cohort selection while allowing clinical judgment in practice.

### 3.6. Contemporary Refinements and Ongoing Challenges

The recently proposed 2023 ACR/EULAR classification criteria represent further evolution, incorporating microvascular thrombosis, cardiac valve abnormalities, and thrombocytopenia among clinical domains while refining laboratory criteria to address seronegative APS and variable assay performance [59].

Despite these advances, significant challenges persist, including inter-laboratory variability in LA testing, and the need for a better definition of seronegative APS. Combining anti-β2GPI domain I and aPS/PT testing with improved external quality assurance programs may help address these issues [60].

A two-tier scoring system has been introduced, distinguishing moderate positivity (40–79 units) from high positivity (≥80 units). However, discrepancies between ELISA and chemiluminescence immunoassay (CLIA) measurement methods pose challenges to diagnostic consistency. Additionally, the shortage of Harris Standards underscores the need for further standardization of measurement methods. To address this issue, the use of “standard antibodies/monoclonal antibodies,” such as HCAL or EY2C9 as mentioned earlier [46,47], should be thoroughly considered. Future research is essential to validate the effectiveness and reliability of these updated criteria [61].

The 2023 ACR/EULAR APS criteria use a weighted clinical and lab scoring system and improved specificity. In 126 Korean patients classified by the 2006 revised Sapporo criteria [54], 84.9% met the new criteria. Concordance was highest in VTE (100%), moderate in arterial thrombosis (76.4%), and lowest in obstetric APS (45.5%), likely due to stricter obstetric requirements (e.g., pre-eclampsia, placental insufficiency) [62].

In conclusion, while the 2023 ACR/EULAR criteria mark a notable step forward in standardizing APS classification for research purposes, their current limitations in clinical sensitivity and applicability highlight the need for careful consideration in real-world settings. Balancing research specificity with clinical relevance will be essential to ensure that patients with genuine APS manifestations receive an appropriate diagnosis and care. Future updates should aim to refine the criteria by incorporating real-world evidence and addressing the identified gaps to enhance both research utility and patient outcomes [63,64].

## 4. Pathogenesis

### 4.1. Signaling Pathways and Inflammatory Responses

Cell activation is a critical mechanism in the pathogenesis of APS [33]. aPL target β2GPI and this interaction triggers a cascade of pro-inflammatory and pro-coagulant responses. When aPL bind to β2GPI on the surface of endothelial cells, monocytes, or platelets, they initiate signaling pathways that shift these cells from an anti-thrombotic to a pro-thrombotic state. Specifically, aPL/β2GPI complexes stimulate TF expression, a major initiator of the coagulation system, and promote the production of thromboxane, a potent vasoconstrictor and platelet activator.

The p38 MAPK pathway plays a central role in mediating these effects. Binding of aPL/β2GPI activates p38 MAPK, leading to downstream events such as the activation of NF-κB, which translocates to the nucleus and induces the expression of TF, pro-inflammatory cytokines (e.g., TNF-α, IL-1), and adhesion molecules [16,17,18,64,65,66,67,68,69]. These changes amplify the inflammatory and coagulation responses. Inhibition of p38 MAPK using specific inhibitors like SB203580 has been shown to reduce TF expression, thromboxane production, and cytokine release, highlighting its potential as a therapeutic target. This pathway provides insight into the molecular mechanisms driving APS-related thrombosis.

Accumulating evidence demonstrates that lipid rafts serve as critical signaling platforms in APS pathogenesis [70,71]. Recent investigations have revealed that anti-β2-GPI antibodies interact with the LRP8/ApoER2 receptor within cholesterol and glycosphingolipid-enriched membrane microdomains, triggering downstream phosphorylation events that culminate in endothelial dysfunction. Notably, methyl-β-cyclodextrin (MβCD), an agent that selectively disrupts lipid raft architecture by cholesterol depletion, effectively prevents anti-β2-GPI antibody-induced LRP8 and Dab2 phosphorylation as well as the consequent suppression of endothelial nitric oxide synthase (e-NOS) activation and nitric oxide production. These findings establish lipid raft disruption as a novel therapeutic target and suggest that cyclodextrin-based raft-targeting strategies may provide innovative approaches to complement traditional anticoagulant therapies in APS management.

Tang et al. demonstrated that anti-β2GP1 antibodies activate the mTORC2/Akt pathway in APS platelets, inducing phosphorylation of Akt-S473 and SIN1-T86, with genetic deletion of platelet SIN1 protecting mice from anti-β2GP1-induced thrombosis in three experimental models [72]. In renal endothelial cells, Canaud et al. showed that APS patients exhibited mTOR pathway activation (evidenced by S6 and Akt-S473 phosphorylation), and rapamycin treatment in transplant recipients prevented mTOR activation and reduced endothelial hyperplasia [73], suggesting mTOR inhibition as a therapeutic approach.

Complement activation plays a central role in the pathophysiology of APS, APLs, particularly anti-β2GPI antibodies activate complement pathways, including the classical and alternative pathways, leading to the generation of components like C3 and C5 [19,20,21]. This activation results in inflammation, endothelial cell damage, and procoagulant activity. The interplay between complement and coagulation systems is critical in APS, as complement activation can amplify thrombosis through the production of C5a, which recruits neutrophils and induces TF expression. Additionally, β2GPI has been identified as a natural regulator of complement activation, and its conformational changes can influence complement activity [74,75].

### 4.2. The Two-Hit Hypothesis

The “two-hit” hypothesis is a central framework to explain thrombotic events in APS [33,76,77,78,79]. The first hit comprises a prothrombotic priming state driven by aPL, notably anti-β2GPI, aCL, and LA. APL bind to β2GPI or prothrombin on phospholipid surfaces and activate endothelial cells, monocytes, and platelets. This leads to upregulation of TF, expression of adhesion molecules, perturbation of the annexin A5 anticoagulant shield, interference with the protein C pathway, and attenuation of fibrinolysis via increased PAI-1. Complement engagement—particularly via classical and alternative pathways—further contributes to endothelial activation and a proinflammatory, procoagulant milieu. Collectively, these mechanisms establish a primed state in which thrombin generation is facilitated but may remain clinically silent in the absence of triggers.

The second hit is provided by an external or endogenous stimulus that tips the hemostatic balance toward overt thrombosis. Common triggers include infections, surgery, trauma, immobilization, estrogen exposure, smoking, and inflammatory flares. These stimuli amplify endothelial dysfunction, enhance neutrophil extracellular trap (NET) formation, and intensify complement activation (e.g., C3/C5 cleavage), thereby accelerating thrombin generation and stabilizing fibrin. Animal models and ex vivo systems show that aPL alone seldom suffices to induce thrombosis; however, when combined with inflammatory stimuli such as lipopolysaccharide or venous stasis, thrombus formation becomes robust, underscoring the necessity of a second hit. Clinically, this paradigm explains why many aPL-positive individuals remain asymptomatic and why risk mitigation—anticoagulation, hydroxychloroquine, statins, and meticulous management of intercurrent triggers—reduces event rates by attenuating either first-hit priming or second-hit amplification. The concept also informs perioperative and infection-related prophylaxis and supports therapeutic strategies targeting platelets, tissue factor, and complement, linking mechanistic understanding directly to prevention and treatment of APS-related thrombosis.

### 4.3. NETs in APS Pathogenesis

NETs are crucial contributors to both thrombotic and obstetric manifestations of APS [79]. NETs consist of DNA-histone scaffolds that promote thrombus formation, activate coagulation cascades, and inactivate natural anticoagulants. APS patients exhibit elevated circulating NET markers, including cell-free DNA and myeloperoxidase-DNA complexes, along with impaired DNase-mediated NET degradation [80]. Anti-NET antibodies have been identified in some APS patients, indicating a failure of normal homeostatic NET clearance mechanisms and suggesting additional biomarker and therapeutic targets [81]. These findings position NETs as both pathogenic mediators and potential therapeutic targets in APS management [82].

### 4.4. Interferon Signatures in APS

**Figure 2:**
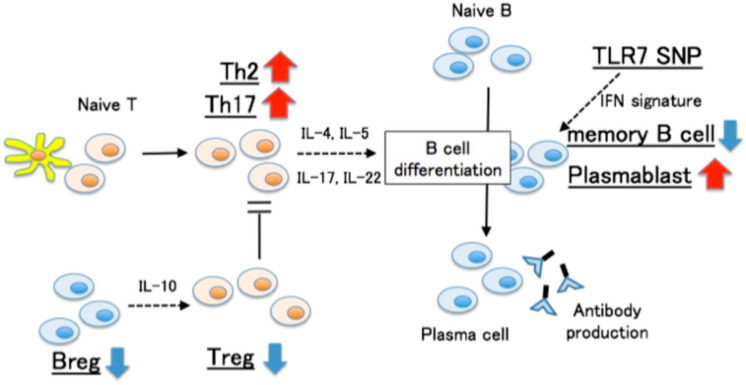
Immune cell subsets in APS patients. [Adapted from reference [23]].

SLE and APS share significant clinical and immunological features. Hisada et al. [23] showed that, compared with healthy subjects, patients with APS had increased frequencies of Th2 cells, Th17 cells, and plasmablasts, along with decreased frequencies of regulatory T cells, memory B cells, and regulatory B cells. Genetic analysis further revealed that patients carrying the TLR7 rs3853839 GG risk genotype had a significantly increased plasmablast frequency (Figure 2). Notably, this immune subset profile is highly similar to that observed in SLE. Elevated type I IFN signatures have been observed in APS and SLE-APS patients, even in subclinical stages, suggesting a role in early disease progression [83,84].

Recent transcriptomic analysis using RNA sequencing identified interferon-regulated genes (IRGs) such as IFI44, IFI44L, RSAD2, and MMP8 as key drivers of thrombotic APS, with significantly elevated expression compared to healthy controls [85]. Machine learning models based on IRGs demonstrated high diagnostic accuracy (86%), highlighting their potential as biomarkers. High-risk groups, including triple-aPL positive and recurrent thrombosis patients, showed pronounced IRG expression, while complement-related genes (C4A and C4B) were also upregulated in triple-aPL positive individuals, implicating the complement system in APS pathogenesis. Subgroup analyses revealed distinct molecular pathways, with venous thrombosis patients showing higher IRG abundance and arterial thrombosis patients displaying neutrophil-related gene expression. These findings emphasize the central role of IRGs and the complement system in thrombotic APS pathogenesis, offering promising avenues for personalized treatment strategies and future research.

### 4.5. A Comprehensive GWAS Study

A genome-wide association study (GWAS) analyzed 5485 individuals of European ancestry, including 482 primary APS patients, to identify genetic susceptibility loci and provide insights into the disease’s pathophysiology [86]. The study revealed two significant loci: STAT1-STAT4 (rs11889341) and HLA-DRA (rs9269041). These loci are associated with immune system regulation, with HLA-DRA linked to overexpression of HLA class II genes in blood, vascular, and nervous tissues, suggesting a role in primary APS pathogenesis. Additionally, 34 suggestive loci were identified, including ESR2 (estrogen receptor 2) and HGF (hepatocyte growth factor), both implicated in thrombosis.

The study also highlighted genetic overlaps between APS and other immune-mediated diseases such as neuromyelitis optica, Sjögren syndrome, and systemic sclerosis. Functional analyses revealed that PAPS-associated variants influence immune-related pathways, including T-cell activation and cell adhesion, emphasizing the immune system’s role in disease development. Furthermore, genetic risk scores (GRS) indicated population-level variability, with the highest risk observed in East Asian and African populations.

These findings provide valuable insights into the genetic underpinnings of APS and its shared mechanisms with other IMDs, paving the way for future therapeutic strategies.

## 5. Conclusions: Toward Individualized APS Care

APS has evolved from the identification of aPLs and β2GPI into a cohesive model of immunothrombosis driven by complement activation, NET biology, and cellular signaling across endothelial, platelet, and innate immune pathways. Diagnostic criteria and laboratory tools have matured considerably, yet persistent heterogeneity and assay standardization challenges continue to hinder uniform risk assessment and therapeutic decisions.

Looking forward, precision diagnostics anchored in harmonized LA assays, expanded testing for anti-domain I and aPS/PT, and multimodal biomarkers—spanning genetics, immune profiling, and advanced imaging—will enable more accurate stratification of thrombotic and obstetric risk. In parallel, targeted therapeutics that interrupt key pathogenic cascades (complement, NET formation, tissue factor signaling, etc.) hold promise for reducing thrombotic recurrence, microvascular complications, and pregnancy morbidity beyond what is achievable with anticoagulation alone.

Achieving individualized APS care will require high-quality, biomarker-guided randomized trials, integration of real-world evidence through international registries, and iterative refinement of guidelines that reflect diverse patient trajectories. With coordinated efforts in assay harmonization, mechanistic therapeutics, and data-driven implementation, the coming decade is poised to translate pathobiological insights into personalized, outcome-focused APS management.

## Data Availability

No new data were created or analyzed in this study.
